# 

**Published:** 1877-12

**Authors:** 


Vol. 24. DECEMBER, 1877. No. 12.

TWENTY-FOURTH YEAR.

### HALL'S

### Journal of Health.

PUBLISHED AT BIBLE HOUSE,
THIRD AVENUE, CORNER OF NINTH STREET,
NEW YORK.

E. H. GIBBS, M.D., Editor.

AGENTS FOR THE TRADE:
AMERICAN NEWS COMPANY, 121 NASSAU STREET.
NEW YORK NEWS COMPANY, 18 BEEKMAN STREET.

Terms, $1.50 a Year, or $1.00 for Eight Months. Postage paid.
SINGLE NUMBER, 15 CENTS.

Couch & Johnston, Printers, 11 Frankfort St., New York.

